# Down-Regulation of *Inpp5e* Associated With Abnormal Ciliogenesis During Embryonic Neurodevelopment Under Inositol Deficiency

**DOI:** 10.3389/fneur.2021.579998

**Published:** 2021-05-19

**Authors:** Huixuan Yue, Shen Li, Jiaxing Qin, Tingting Gao, Jianjun Lyu, Yu Liu, Xiuwei Wang, Zhen Guan, Zhiqiang Zhu, Bo Niu, Rugang Zhong, Jin Guo, Jianhua Wang

**Affiliations:** ^1^Beijing Municipal Key Laboratory of Child Development and Nutriomics, Capital Institute of Pediatrics, Beijing, China; ^2^Graduate School of Peking Union Medical College, Beijing, China; ^3^Beijing Key Laboratory of Environment and Viral Oncology, College of Life Science and Bioengineering, Beijing University of Technology, Beijing, China; ^4^Department of Pathology, InnoStar Bio-Tech Nantong Co., Ltd., Nantong, China

**Keywords:** phosphoinositide 5-phosphatase, gene expression, cilia, embryonic development, neural tube defects, inositol

## Abstract

The inositol polyphosphate-5-phosphatase E (*Inpp5e*) gene is located on chromosome 9q34.3. The enzyme it encodes mainly hydrolyzes the 5-phosphate groups of phosphatidylinositol (3,4,5)-trisphosphate (PtdIns (3,4,5) P3) and phosphatidylinositol (4,5)-bisphosphate (PtdIns (4,5)P2), which are closely related to ciliogenesis and embryonic neurodevelopment, through mechanisms that are largely unknown. Here we studied the role of *Inpp5e* gene in ciliogenesis during embryonic neurodevelopment using inositol-deficiency neural tube defects (NTDs) mouse and cell models. Confocal microscopy and scanning electron microscope were used to examine the number and the length of primary cilia. The dynamic changes of *Inpp5e* expression in embryonic murine brain tissues were observed during Embryonic Day 10.5–13.5 (E 10.5–13.5). Immunohistochemistry, western blot, polymerase chain reaction (PCR) arrays were applied to detect the expression of *Inpp5e* and cilia-related genes of the embryonic brain tissues in inositol deficiency NTDs mouse. Real-time quantitative PCR (RT-qPCR) was used to validate the candidate genes in cell models. The levels of inositol and PtdIns(3,4) P2 were measured using gas chromatography-mass spectrometry (GC-MS) and enzyme linked immunosorbent assay (ELISA), respectively. Our results showed that the expression levels of *Inpp5e* gradually decreased in the forebrain tissues of the control embryos, but no stable trend was observed in the inositol deficiency NTDs embryos. *Inpp5e* expression in inositol deficiency NTDs embryos was significantly decreased compared with the control tissues. The expression levels of *Inpp5e* gene and the PtdIns (3,4) P2 levels were also significantly decreased in the inositol deficient cell model. A reduced number and length of primary cilia were observed in NIH3T3 cells when inositol deficient. Three important cilia-related genes (*Ift80, Mkks, Smo*) were down-regulated significantly in the inositol-deficient NTDs mouse and cell models, and *Smo* was highly involved in NTDs. In summary, these findings suggested that down-regulation of *Inpp5e* might be associated with abnormal ciliogenesis during embryonic neurodevelopment, under conditions of inositol deficiency.

## Introduction

The *Inpp5e* gene encodes the inositol polyphosphate-5-phosphatase E (INPP5E), a 72 kDa protein which mainly hydrolyzes PtdIns (3,4,5)P3 and PtdIns(4,5)P2. These compounds are key mediators in signaling pathways that influence a large range of cellular functions (1, 2). Subcellular locations of the INPP5E protein include the cilium axoneme, neural cytoplasm, cytoskeleton, and golgi apparatus. Previous results have indicated that mutations in *Inpp5e* lead to neural malformations, including Joubert syndrome (JBTS), Behr syndrome and micropenis (MORM) syndrome (3–5). Knockout of the *Inpp5e* gene (*Inpp5e*^−/−^) in mice resulted in embryonic and early postnatal death with a phenotype that recapitulates JBTS, containing NTDs, polycystic kidneys and polydactyly (6). A null allele of *Inpp5e* caused abnormal Shh response, which played a critical role in the intermediate region along the dorso-ventral (D-V) axis of the developing neural tube at E 10.5 (7). In a previous study, we found that the expression of *Inpp5e* decreased during a normal course of embryonic development, but no obvious trend was observed in methotrexate (MTX)-induced NTDs mice embryos, and that the expression levels of *Inpp5e* in NTDs embryos was significantly lower than the controls at E 11.5 (8). These studies suggested that changes in the expression pattern of *Inpp5e* were involved in the development of NTDs. However, the underlying mechanism by which the *Inpp5e* gene regulates neural tube closure remains poorly understood.

NTDs result from the failure of neural tube closure during weeks 3–5 after fertilization in humans (9), and represent a series of severe and common malformations of the central nervous system affecting 0.7–3%0 live births (10, 11). There is a complicated etiology that involves numerous genetic and environmental factors (10). Accumulating evidence has suggested that maternal inositol deficiency during pregnancy contributes to the development of NTDs in the offspring (12–14). Knockout of genes that encode key-enzymes involved in inositol metabolism, such as *Inpp5e*, type Iγ phosphatidylinositol 4-phosphate-5-kinase (*PIPKI*γ) or inositol 1,3,4,triphosphate 5/6-kinase (*Itpk1*) could induce NTDs (15, 16). However, the mechanisms of coordinate effects between inositol metabolism and its key-enzymes on the development of NTDs remain elusive.

Primary cilia, an antenna-like structure, protrude from the surface of cells, can sense extracellular mechanical, chemical stimulation, and mediate signal transduction in neural pathways. Structural and functional defects of primary cilia are related to malformations of the neural tube (5, 17, 18). A growing number of evidence has demonstrated that primary cilia are crucial for embryonic neural development, mainly through modulating cell cycle progression, Wnt signaling and Hedgehog signaling (10, 17). NTDs are observed in some mouse mutants with impaired primary cilia, including mutants for some intraflagellar transport proteins, the retrograde motor subunit mutants, and some other genes whose mutations lead to abnormal cilia (17). Nevertheless the regulatory mechanism of primary cilia during neurodevelopment is still unclear.

Mutations in *Inpp5e* affect the biogenesis and stabilization of cilia (1, 19–22). Mutations in the phosphatase domain could damage the 5-phosphatase activity, which changes the ratios of cellular phosphatidyl inositol (PtdIns) species. These studies suggest that the involvement of the phosphatidylinositol signaling pathway in primary cilia has an important role in mediating neurodevelopment (1, 19, 21). Apart from cranial NTDs, *Inpp5e*-knockout mice showed characteristics of ciliopathies, for instance polydactyly and polycystic kidneys (23). Mutated *Inpp5e* has been reported in ciliopathies: MORM Syndrome and JBTS (5, 16). Given the substrate and products of *Inpp5e* both are important inositol derivatives, we speculated that inositol metabolism plays an important role in the process of neural tube closure, through regulating the expression of *Inpp5e* and thus affecting the ciliogenesis.

In this study, we investigated the role of *Inpp5e* in ciliogenesis during embryonic neurodevelopment under inositol deficiency, using the inositol deficiency NTDs mouse model and *in vitro* cultured cells. The expression of *Inpp5e* and cilia-related genes were detected during ciliogenesis in mouse brain tissues under inositol deficiency. The results were validated in NIH3T3 cells *in vitro*. This study was undertaken to provide further information on the role of the *Inpp5e* gene in ciliogenesis during embryonic neural development and a research basis for the pathogenesis, prevention and treatment of NTDs.

## Materials and Methods

### Animals and Li_2_CO_3_ Treatment

C57BL/6 mice, 7-9 weeks old, weighing 19–22 g, specific pathogen free (SPF) grade, were purchased from Beijing Vital River Laboratory Animal Technology Co., Ltd. Mice housed in animal rooms under controlled temperature of 22 ± 2 °C, and humidity of 50 ± 10%, with a 12 hours (h) light/dark cycle, were given food and water *ad libitum*. Female and male mice were mated overnight (from 6:00 p.m. to 6:00 a.m.), the presence of a vaginal plug was detected the next morning, which was considered to be E 0.5 when present.

The pregnant mice, treated with an intraperitoneal injection of Li_2_CO_3_ (350 mg/kg) at E 7.5 to induce an inositol deficiency mouse model of NTDs, were used as the experimental group (24). The control group was given the same dose of 0.9% NaCl at E 7.5. Pregnant mice were euthanized by cervical dislocation, and embryos were examined under a dissecting microscope at E 10.5 to E 13.5. Some embryos were placed in cold 4% paraformaldehyde for tissue processing, sectioning, and subsequent examination by light microscopy. For the molecular biology experiments, brain tissues of normal embryos from the control group were isolated and pooled as control samples, and brain tissues of NTDs embryos from the Li_2_CO_3_ group were isolated and pooled as embryonic development defect samples. Each sample represented a pool of two or three embryonic brain tissues from one litter. To detect the plasma inositol concentration of pregnant mice, blood samples were collected from the caudal vein at 0, 4, 8, 16, 24, and 48 h after intraperitoneal injection of Li_2_CO_3_ (350 mg/kg). All of the samples were stored at −80°C.

### Immunohistochemistry

The fixed tissues were dehydrated and embedded in paraffin following routine methods. After emoving paraffin, sections were immersed in phosphate buffered saline with 0.05% Tween-20 (PBS-T), and then blocked with 3% peroxide-methanol for 60 minutes (min), at room temperature for endogenous peroxidase ablation. Sections were incubated with the antibody against INPP5E overnight at 4°C, and then with secondary antibody for 60 min at 37°C. Slides were counterstained using hematoxylin, dehydrated in sequential ethanol and xylene washes and mounted using distyrene-plasticizer-xylene (DPX). The number and proportion of positive cells were observed and recorded under a light microscope.

### Detection of Inositol Levels

Inositol levels were measured as described previously (18). Briefly, plasma samples (30 μL) collected from pregnant mice or cell lysates (30 μL) from NIH3T3 cells were mixed with anhydrous ethanol (10 mL), and dried using the rotary evaporator at 71°C. Then 5 mL N, N-dimethylformamide (N-DMF) /hexamethyldisilazane (HMDS) /Trimethylchlorosilane (TMCS) (8: 2: 1, v / v / v) was added and dried again at 71°C for an hour. After adding hexanes (5 mL) and saturated NaCl solution (10 mL) samples were vortexed for 2 min and centrifuged at 6, 500 rotations per min (rpm) for 5 min. The supernatants were evaporated to dryness under nitrogen at 40°C. Residues were resuspended with hexanes (1 mL) and inositol levels were analyzed by the gas chromatography-mass spectrometry system (Agilent Technologies, Germany).

### Western Blot

Protein samples were extracted from embryonic brain tissues or cells in a RIPA (RadioImmunoPrecipitation Assay) buffer with a protease inhibitor cocktail (Sangon Biotech, China). Six pooled control embryonic brain tissues and 6 pooled NTDs embryonic brain tissues at E 10.5–13.5 were tested by western blot. Primary antibodies against INPP5E (1:1000, Proteintech, USA) and glyceraldehyde-3-phosphate dehydrogenase (GAPDH, 1:1000, Santa Cruz Biotechnology, USA) and secondary anti-rabbit horseradish-peroxidase-conjugated antibodies (1:5, 000, Proteintech, USA) were used. Blots were detected by chemiluminescence using the SuperSignal West Pico Chemiluminescence Substrate (Thermo Fisher, USA). Image analysis was performed using a gel image processing system.

### Cell Culture

NIH3T3 cells were obtained from American type culture collection (ATCC) and cultured in high glucose Dulbecco modified Eagle medium (DMEM, Gibco, USA) with 10% fetal bovine serum (FBS, Gibco, USA) and 100 × penicillin-streptomycin (Thermo Fisher, USA). Cells were maintained at 37°C in a humidified atmosphere with 5% CO_2_. NIH3T3 cells were passaged at 80% confluence with 0.25% trypsin-ethylene diamine tetraacetic acid (EDTA, Gibco, USA) every other day.

### MTT Assay

For the MTT [3-(4, 5-dimethylthiazol-2-yl)-2, 5-diphenyltetrazolium bromide] assay, NIH3T3 cells were seeded and cultured into 96-well plates for 24 h, and then treated with different doses (0, 0.5, 1, 2, 4, 6, 8, 12, 18, and 36 mM) of Li_2_CO_3_ for 24 h. Cells were incubated with medium containing 5 mg/mL MTT (Sigma, USA) for 4 h, dimethyl sulfoxide (DMSO, 150 μL, Solarbio, China) was added into each well to stop the reaction. The absorbance of the plate was checked at the wavelength of 490 nm by a microplate reader (Thermo Fisher Scientific, USA).

### Lipid Extraction and ELISA

Lipid extractions were performed as previously described (25). After a final extraction step, 700 μL liquid extracts were transferred into a 1.5 mL EP tube for PtdIns(3,4)P2 detection. After chloroform was removed in vacuum desiccator, lipid extracts were resuspended in 3% Protein Stabilizer with 125 μL PBS-Tween. The protein stabilizer was offered by ELISA kits (Echelon Biosciences, USA). ELISA was carried out following the manufacturer's instruction.

### Immunofluorescence

The serum-starved NIH3T3 cells growing on glass coverslips were fixed with 1 mL 4% paraformaldehyde (15 min), permeabilized with 2 mL 0.3% Triton X-100 (10 min), and blocked with 5% bovine serum albumin (BSA) for 1 h. After incubated with mouse anti-acetylatedα-tubulin (1:100, Abcam, USA) for 3 h at room temperature, cells were exposed to Alexa Fluor488-conjugated secondary antibody (1:200, Abcam, USA) for 1 h at 37°C. 4',6-diamidino-2-phenylindole (DAPI, Solarbio, China) was used to stain the nuclei, and photomicrographs were obtained with an inverted confocal microscope (Leica, Germany). The cells stained with acetylated tubulin antibody were considered as ciliated cells. The number of primary cilia was observed by the naked eye. Cilia length was measured using straight- or segmented-line tool for selection of fluorescent signals of ciliary marker in maximum Z intensity projected images using Image J.

### Scanning Electron Microscopy

The serum-starved NIH3T3 cells were fixed in 2.5% glutaraldehyde for 2 h and post-fixed in 1% OsO4 for 1 h at room temperature, dehydrated was performed in a gradient series of ethanol (30%, 50%, 70%, 95%, 2 times for 10 min each, and 3 times with 100%). After incubation with tetramethylsilane solution (10 min each, 3 times in total), cell images were captured on SU-8010 scanning electron microscope (Hitachi, Japan). For each group, twenty fields were randomly chosen, cilia number and length were examined, and statistical differences were calculated. The number of primary cilia was observed by the naked eye. Cilia length was measured using the scale included in the images taken.

### RNA Extraction

Total RNA was extracted using the RNeasy® Micro Kit (Qiagen, Germany). The purity and concentration was analyzed by a UV-Vis spectrophotometer (NanoDrop 2000, USA) in 2 μl samples. All samples exhibited 260/280 nm and 260/230 nm absorbance ratio between 1.8 and 2.1 (average of 1.89). The integrity of RNA was detected using an Agilent 4200 bioanalyser (Agilent Technologies, Germany). RNA Integrity Number for samples was equal to 10.

### PCR Arrays

Total purified RNA (1.5 μg) was used to synthesize cDNA using a SuperScript™ IV First-Strand Synthesis System (Thermo Fisher, USA). The Mouse Primary Cilia RT^2^ Profiler™ PCR Array with SYBR® Green (QIAGEN, Germany) was used to screen the differential expression of 89 cilia-related genes in embryonic murine brain tissues. RT-PCR was performed using the 7900 HT Fast real-time PCR system (Applied Biosystems, USA).

### RT-qPCR

Total RNA extraction and cDNA synthesis of the serum-starved NIH3T3 cells were performed as above. The mRNA expression was tested by RT-qPCR in a 20 μl reaction system containing 10 μl of Eva Green 2x qPCR Master Mix (Applied Biological Materials, Canada), 0.6 μl each of forward and reverse primers (10 μM), 0.4 μl of cDNA, and 8.4 μl of nuclease-free H_2_O (Thermo Fisher, USA). The thermal cycling conditions were 95 °C for 10 min, followed by 35 cycles of 95 °C for 15 seconds (s) and 60°C for 60 s. The expression levels of mRNA were calculated with the 2^−ΔΔCT^ method. Data were normalized to glyceraldehyde 3-phosphate dehydrogenase (*Gapdh*) expression. Primers used were listed in Table 1.

**Table 1:** Primers used for the expression analysis of the cilia-related genes.

**Gene**		**Primer sequence (5^**′**^ to 3^**′**^)**	**Tm (^**°**^C)**	**Product length (bp)**
*Ift80*	Forward	AAGGAACCAAAGCATCAAGAATTAG	58.07	148
	Reverse	AGATGTCATCAGGCAGCTTGAC	60.68	
*Kras*	Forward	GGATATTCTCGACACAGCAGGTCA	62.23	183
	Reverse	ACTAGGACCATAGGCACATCTTCAG	61.68	
*Pkhd1*	Forward	GTGTGGAAGGCGATTATATTGGT	58.87	133
	Reverse	TCTGGGTATGTCTGGTACAGG	58.25	
*Prkca*	Forward	CCAGGAGCAAGCACAAGTT	58.28	142
	Reverse	ATCACACACTGCTTGTGAAC	56.57	
*Smo*	Forward	CAATCGCTACCCTGCGGTTAT	60.54	116
	Reverse	CTGCTCGGCAAACAATCTCTC	59.60	
*Mkks*	Forward	AAGGGCCAAAGAGTTACAGATTC	58.66	104
	Reverse	TCACTGAAGAACTTTTGAGTGGG	58.80	
*Gapdh*	Forward	GAGATTGTTGCCATCAACGACC	60.16	127
	Reverse	CGTTGATGACAAGCTTCCCATT	59.51	

### Statistical Analysis

SPSS 23.0 was used to analyze the data. The measurement data were expressed as mean ± standard deviation (SD). A *Student's t*-test was used to detect statistical differences between two groups of measurements. The comparisons for more than two groups were performed via one-way ANOVA followed by a *post-hoc* Dunnett's test for multiple comparisons. A Chi-square test and Fisher's exact test were used to evaluate differences for the counting data. The results were considered statistically different at *P* < 0.05.

## Results

### Alteration of the *Inpp5e* Expression Patterns and Levels Within Embryonic Brain Tissues in the Inositol-Deficiency NTDs Mouse Model

**Figure 1:**
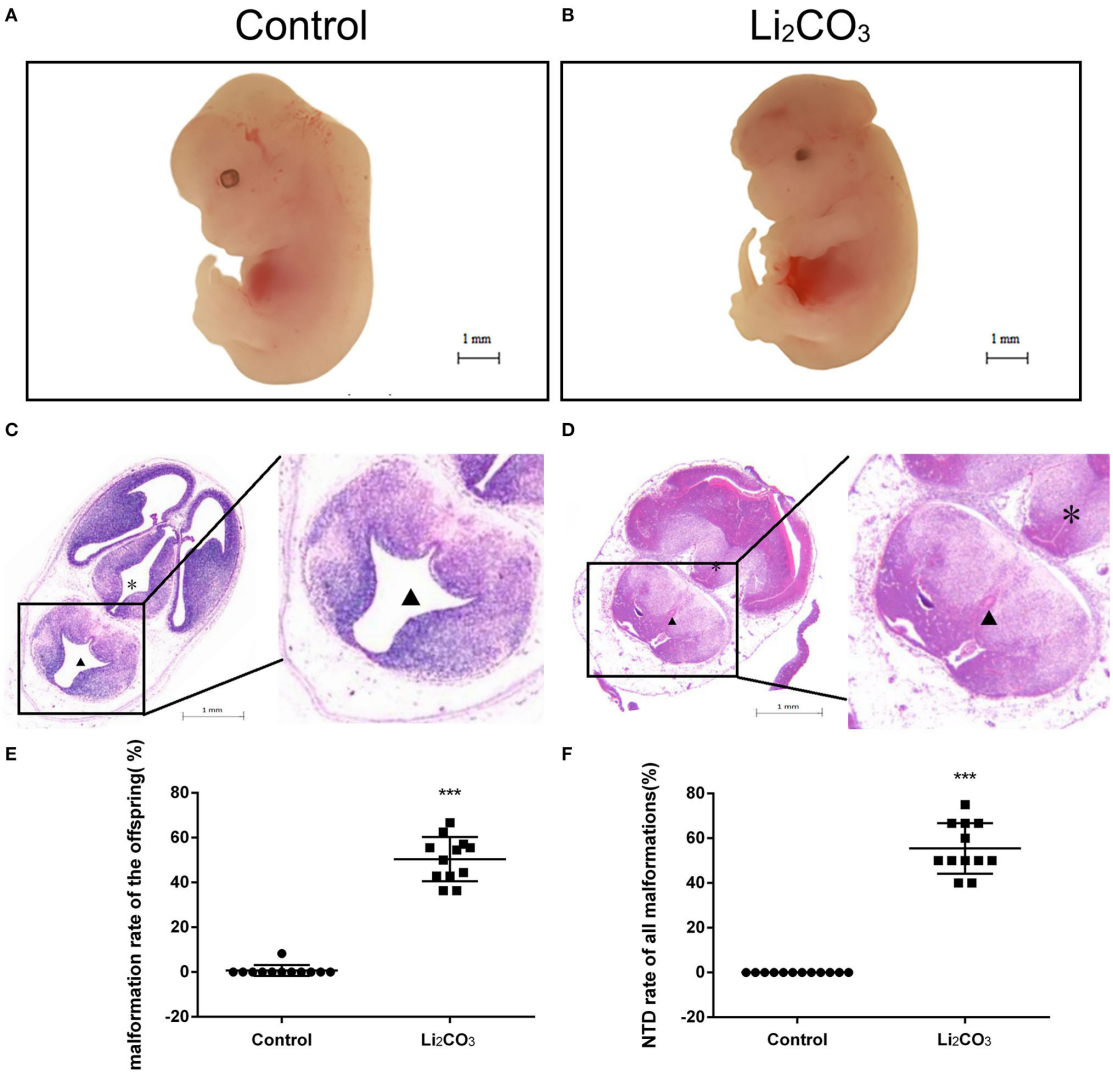
Morphological changes in the NTDs mouse model at E 13.5. **(A)** Control embryos viewed under the dissecting microscope. **(B)** NTDs mouse model embryos viewed under the dissecting microscope. **(C)** Photomicrograph of the light microscopy of the hematoxylin and eosin (H&E)-stained section of mouse embryo brain tissue in the control group (*n* = 6). **(D)** Photomicrograph of the light microscopy of the H&E-stained section of mouse model embryos in the NTDs group (*n* = 6). **(E)** Malformation rate of the offspring in C57BL/6 mice with or without Li_2_CO_3_. Incidence rates were calculated by embryos. **(F)** NTDs rate of all malformations in C57BL/6 mice with or without Li_2_CO_3_. The statistical differences of malformation rate and NTDs rate of all malformations per litter between the control group (*n* = 12) and the NTDs group (*n* = 12) were compared by *student's t*-test. ***P < 0.001 vs. control group. Slice site: *Third ventricle, ^▴^ Fourth ventricle.

The developmental status of embryonic neural tube was observed using stereoscopy in inositol deficiency NTDs mouse model at E 13.5 (Figures 1A,B). The HE staining showed that in the control group, the surrounding cells of the 4^th^ ventricle were densely and homogeneously distributed, with smooth internal and external membranes of the neuroepithelium and regular ventricular wall (Figure 1C), while in the NTDs group, the cephalic plate of the hindbrain was not fused, and the nerve cavity surface was irregular with disordered neuroepithelial cells (Figure 1D). The malformation rate and NTDs rate of all malformations per litter were significantly different in the treated group compared with the control group (*P* < 0.001, Figures 1E,F). Embryos in the control group were well-developed. In the treated group, total malformation rate of the offspring was 50.0%; among them, NTDs accounted for 53.8%. The majority of NTDs phenotypes were encephalocele (Table 2).

**Table 2:** Embryonic phenotypes in C57BL/6 mice induced by 350 mg/kg of Li_2_CO_3_.

**Batch number**	**Group**	**Pregnant mice *n***	**Embryos *n***	**Normal *n* (%)**	**Resorption *n* (%)**	**Total NTDs *n* (%)**	**Encephalocele/NTDs *n* (%)**	**Other malformation *n* (%)**	**Malformation *n* (%)**	**Total NTDs/Malformation *n* (%)**
1	Control	4	36	35 (97.2)	1 (2.8)	0 (0)	0 (0)	0 (0)	1 (2,8)	0 (0)
	Li_2_CO_3_	4	31	17 (54.8)^***^	4 (12.9)^***^	9 (29.0)^***^	7 (77.8)	1^∧^ (32)	14 (45.2)^***^	9 (64.3) ^***^
2	Control	4	30	30 (100)	0 (0)	0 (0)	0 (0)	0 (0)	0 (0)	0 (0)
	Li_2_CO_3_	4	33	14 (42.4)^***^	5(15.2)^***^	9 (27.3)^***^	8 (88.9)	1^#^;2^&^;2^∧^ (15.2)	19 (57.6)^***^	9 (47.4) ^***^
3	Control	4	35	35 (100)	0 (0)	0 (0)	0 (0)	0 (0)	0 (0)	0 (0)
	Li_2_CO_3_	4	40	21 (52.5)^***^	5 (12.5)^***^	10 (25.0)^***^	8 (80.0)	1^§^;3^∧^ (10.0)	19 (47.5)^***^	10 (52.6) ^***^
Total	Control	4	101	100 (99)	1 (0.99)	0 (0)	0 (0)	0 (0)	1 (0.99)	0 (0)
	Li_2_CO_3_	12	104	52 (50.0)^***^	14 (13.5)^***^	28 (26.9)^***^	23 (82.1)	1^§^;1^#^;2^&^;6^∧^ (9.6)^**^	52 (50.0)^***^	28 (53.8) ^***^

∧ *Microphthalmia or adophthalmia*;

# *Cleft lip*;

§ *Development retardation*;

& *Craniofacial deformity*;

** *P < 0.01 vs. control group*;

*** *P < 0.001 vs. control group*.

**Figure 2:**
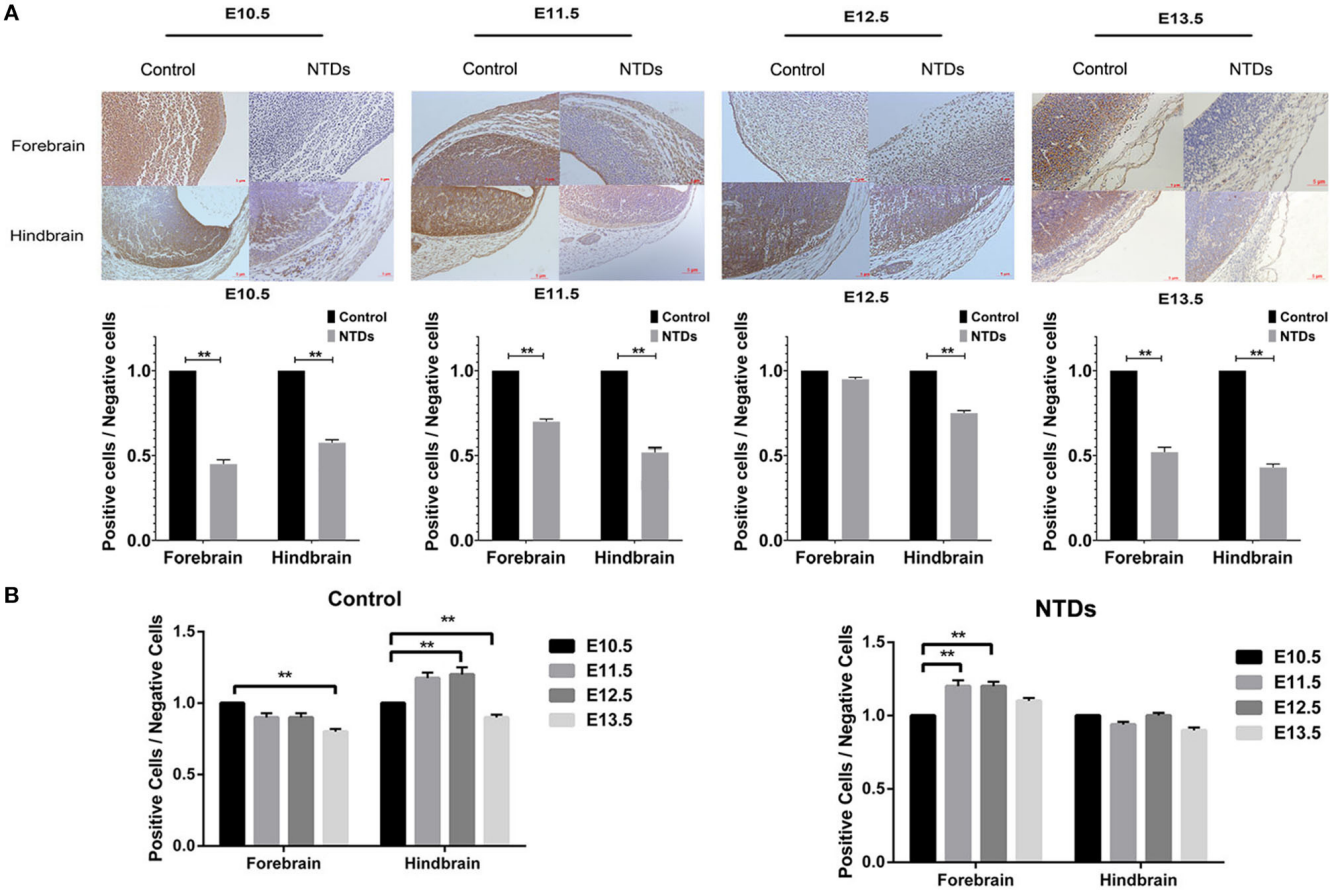
Immunohistochemistry of the protein levels of *Inpp5e* expression in control and NTDs embryonic brain tissues at E 10.5, E 11.5, E 12.5, and E 13.5. **(A)** Immunohistochemistry of the protein expression of *Inpp5e* in embryonic brain tissues from E 10.5 to E 13.5, in forebrain, hindbrain respectively. **P < 0.01 NTDs group (*n* = 6) vs. control group (*n* = 6). **(B)** The dynamic changes of *Inpp5e* expression patterns in embryonic murine brain tissues during E10.5–13.5, in forebrain, hindbrain respectively. **P < 0.01 vs. E 10.5. Each experiment was carried out in triplicate.

**Figure 3:**
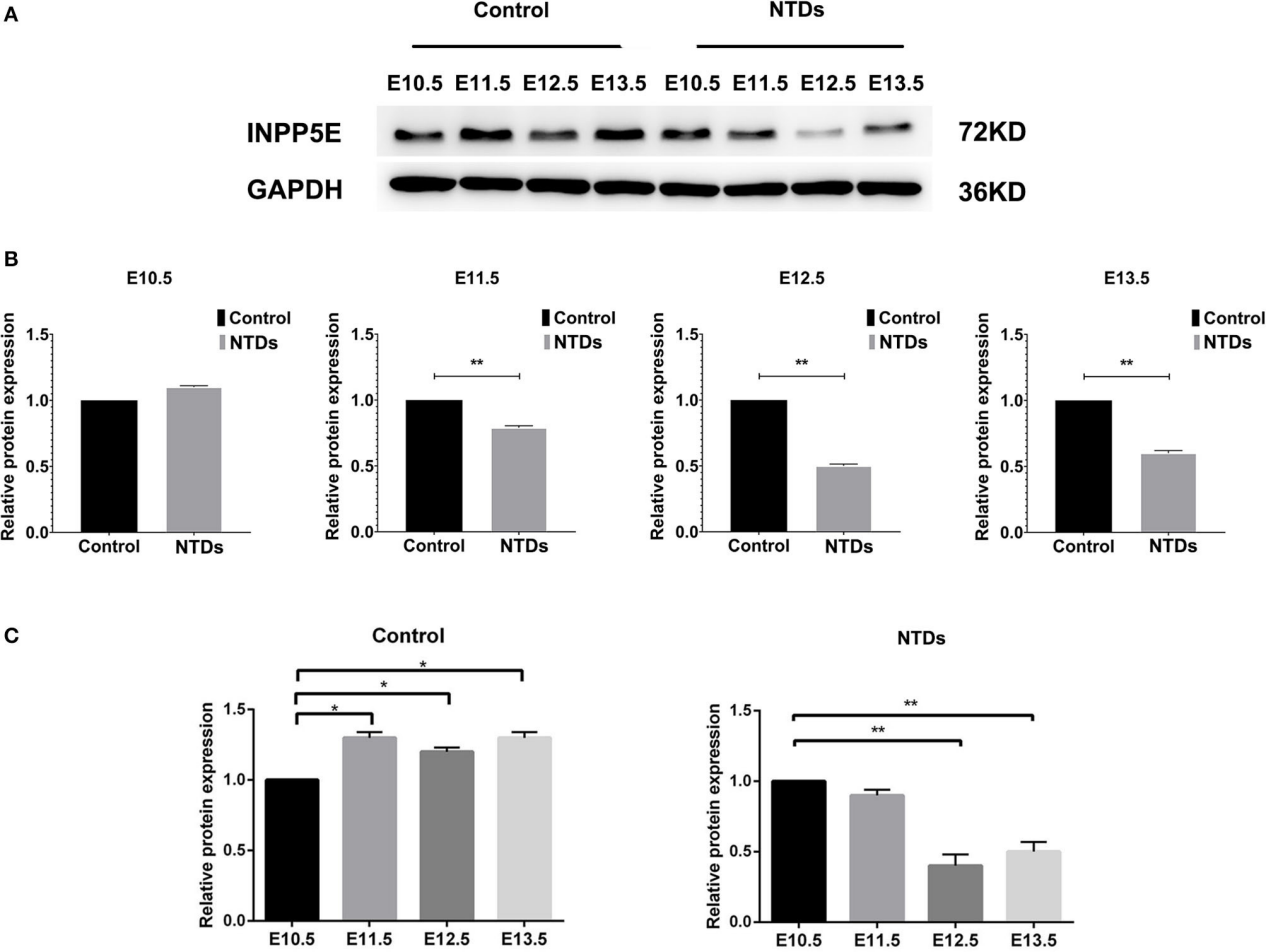
Western blot of the protein levels of *Inpp5e* expression in control and NTDs embryonic brain tissues at E10.5, E 11.5, E 12.5, and E 13.5. **(A, B)** *Inpp5e* expression in embryonic brain tissues from E 10.5 to E 13.5 was determined by western blot. **P < 0.01 NTDs group (*n* = 6) vs. control group (*n* = 6). **(C)** The dynamic changes of *Inpp5e* expression patterns in embryonic murine brain tissues during E 10.5–E 13.5. *P < 0.05, **P < 0.01 vs. E 10.5. Each experiment was carried out in triplicate.

The dynamic expressions of the *Inpp5e* in embryonic murine brain tissue were detected from E 10.5 to E 13.5 (Figures 2, 3). Immunohistochemical staining showed that the expression level of *Inpp5e* were markedly reduced in the brain tissues of the NTDs groups than that in the control group at E 10.5, E 11.5, E 12.5, and E 13.5 (Figure 2A). The *Inpp5e* expression exhibited a decreasing trend in the forebrain of control group (*P* < 0.01); while an increased expression of *Inpp5e* with a peak from E 11.5 to E 12.5 (*P* < 0.01) was observed in NTDs group. There was a peak increasing in the hindbrain of control group, from E 10.5 to E 13.5 (*P* < 0.01), while no obvious trend was observed in the hindbrain of the NTDs group (Figure 2B). Western blot results showed that *Inpp5e* expression was markedly reduced in the whole brain tissues of the NTDs group, compared with control groups at E 11.5, E 12.5, and E 13.5 (Figure 3, *P* < 0.01).

### Decreased *Inpp5e* Expression and Abnormal Primary Cilia in Inositol Deficiency Cells

**Figure 4:**
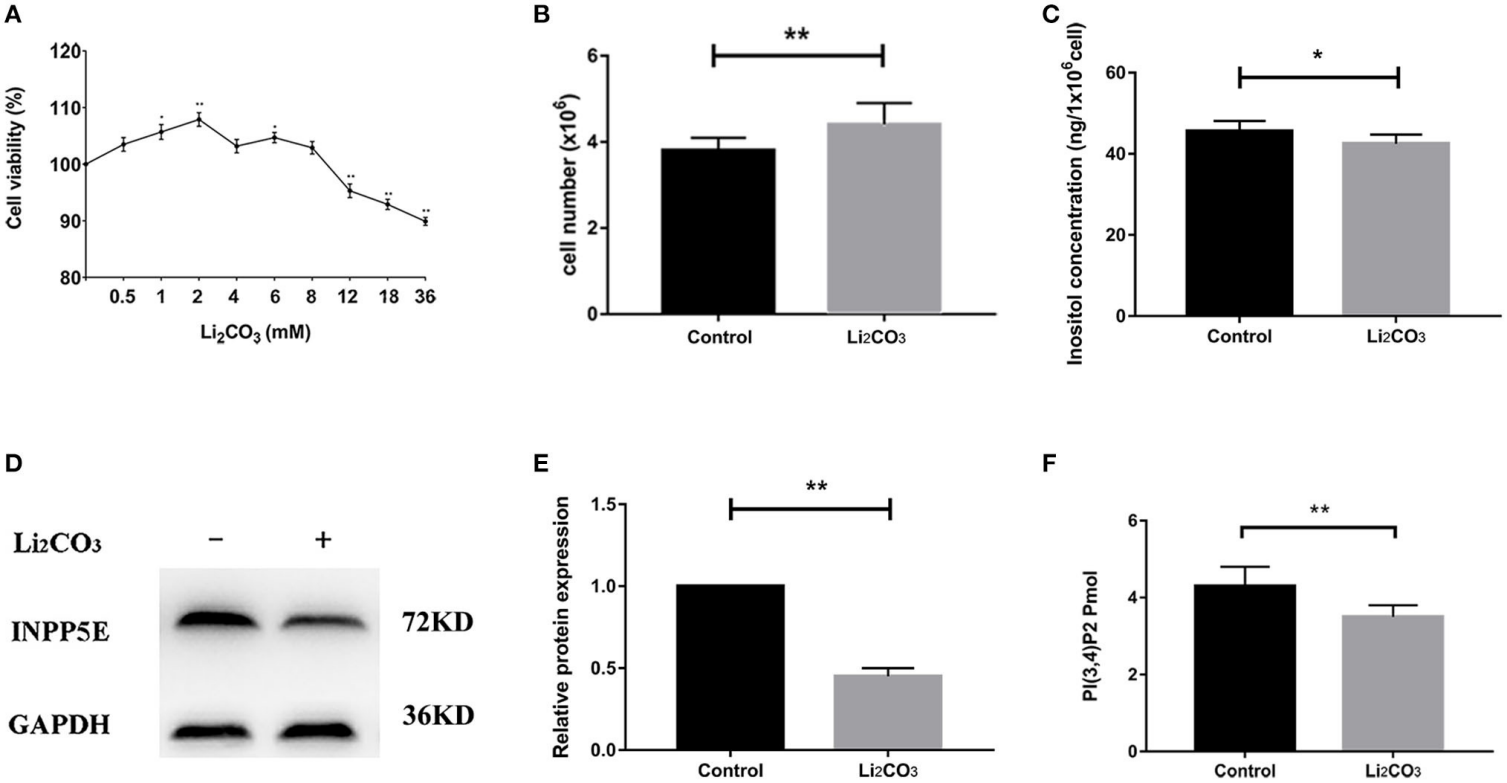
Decreased *Inpp5e* expression in inositol deficiency NIH3T3 cells. **(A)** Cell viability of NIH3T3 cells treated with different doses of Li_2_CO_3_ for 24 h. **(B)** NIH3T3 cells treated with or without 2 mM Li_2_CO_3_ for 24 h, and cell numbers were examined. **(C)** Inositol concentration was measured in NIH3T3 cells treated with or without 2 mM Li_2_CO_3_ for 24 h. **(D, E)** NIH3T3 cells were treated with or without Li_2_CO_3_ for 24 h, and the protein expression of INPP5E were evaluated by western blot. GAPDH were used to confirm equal loading. **(F)** NIH3T3 cells were treated with or without 2 mM Li_2_CO_3_ for 24 h, and PI(3,4)P2 concentration determined. All the analysis in NIH3T3 cells were performed in three independent experiments (*n* = 3). Each experiment was carried out in triplicate. *P < 0.05, **P < 0.01 vs. Control group.

In order to validate the results above and observe the formation of primary cilia, NIH3T3 cells, which can be used to study primary cilia (26), were used to investigate the role of *Inpp5e* gene in ciliogenesis under inositol deficiency. NIH3T3 cells, an immortalized mouse embryo fibroblast cell line, were used to select the optimum concentrations of Li_2_CO_3_. Cell proliferation was analyzed by the MTT assay. A lower concentration (2 mM) of Li_2_CO_3_ promoted the growth of cells while a higher concentration (>10 mM) led to inhibition (Figure 4A). Cell numbers were significantly higher in the 2 mM Li_2_CO_3_ treated group than in controls (Figure 4B, *P* < 0.01). Western blotting revealed a significantly decreased level of INPP5E in NIH3T3 cells following Li_2_CO_3_ treatment (Figures 4D,E, *P* < 0.01), which was consistent with what we observed in the brain tissues of the NTDs mouse model.

**Figure 5:**
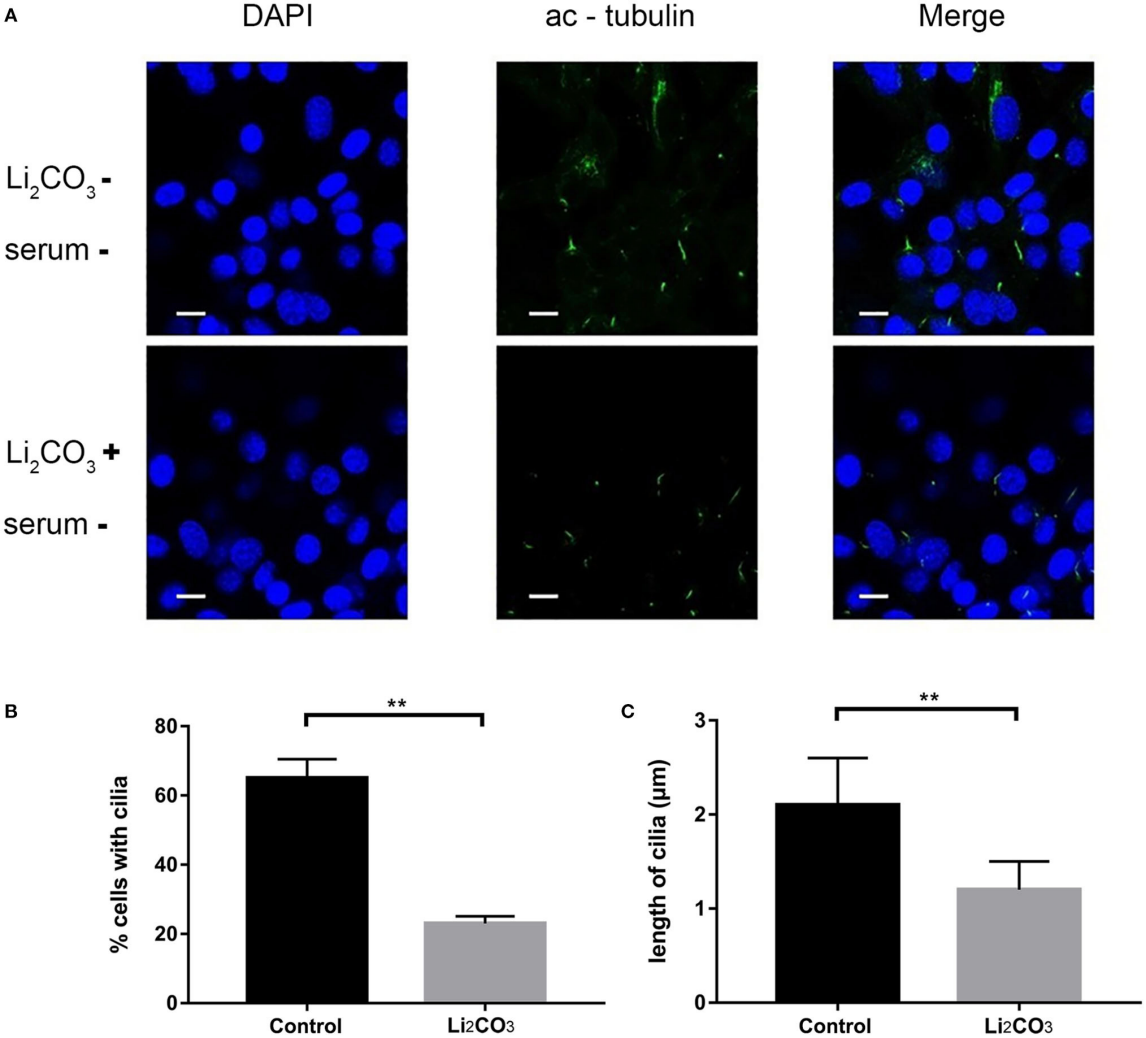
Immunofluorescence of abnormal cilia in inositol deficiency cell model. **(A)** Immunofluorescence representative images of NIH3T3 cells treated with (*n* = 3) or without 2 mM Li_2_CO_3_ (*n* = 3), and stained for primary cilia (green, acetylated tubulin) and nucleus (blue, DAPI). Scale bars, 10 μm. **(B)** Analysis of the percentage of cells with cilia. A total of >100 cells were analyzed per replicate (four replicates). **P < 0.01 vs. controls. **(C)** Quantification of cilia length in NIH3T3 cells. Error bars represent standard error. A total of >100 cells were analyzed per replicate (four replicates). **P < 0.01 vs. control group. Each experiment was carried out in triplicate.

**Figure 6:**
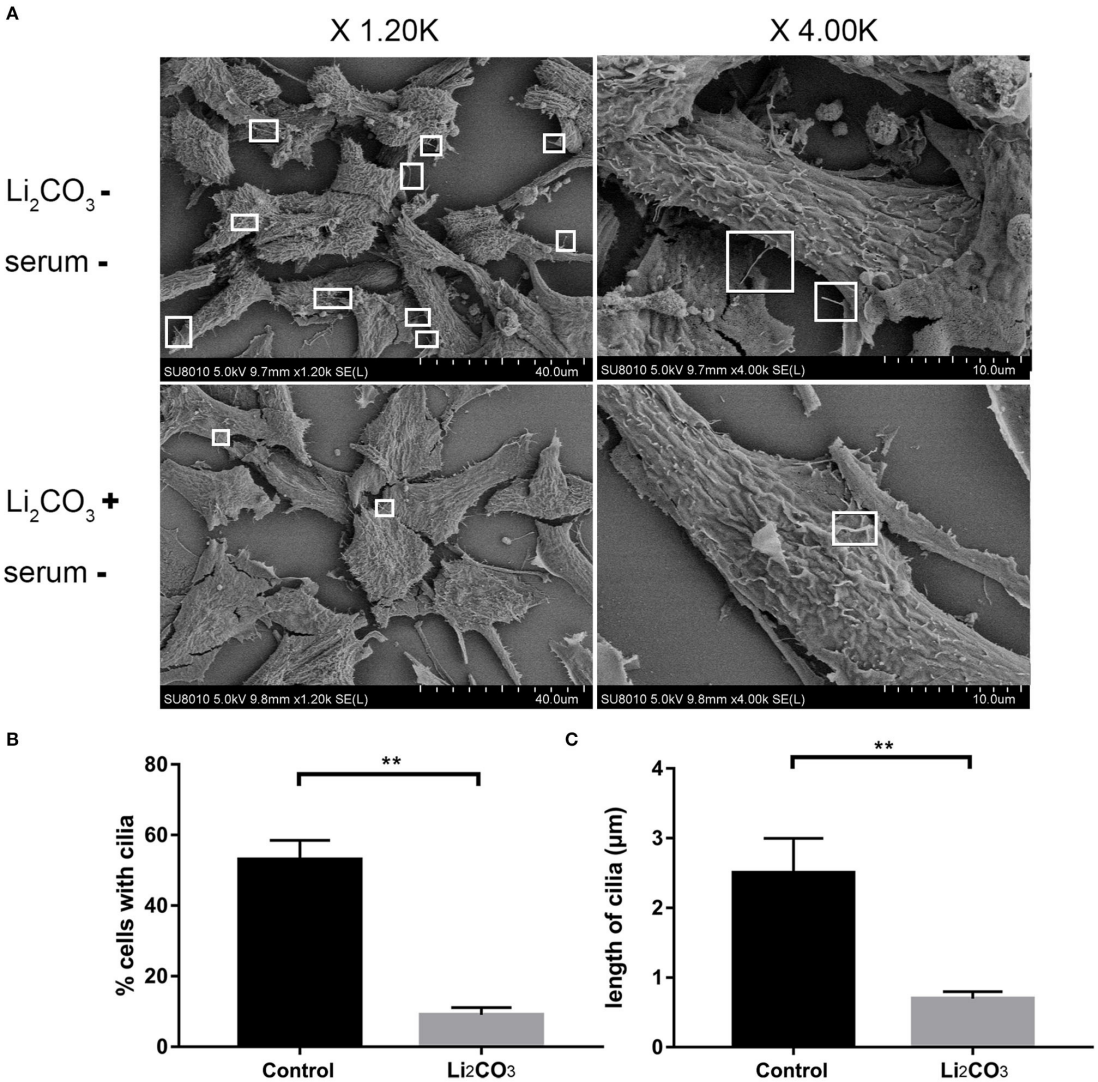
Scanning Electron Microscopy of abnormal cilia in inositol deficiency cell model. **(A)** Scanning electron microscopy views of primary cilia (white boxes) protruding from NIH3T3 cells treated with (*n* = 3) or without 2 mM Li_2_CO_3_ (*n* = 3). **(B)** Analysis of the percentage of cells with cilia. A total of >100 cells were analyzed per replicate (four replicates). **P < 0.01 vs. controls. **(C)** Quantification of cilia length in NIH3T3 cells. Error bars represent standard error. A total of >100 cells were analyzed per replicate (four replicates). **P < 0.01 vs. control group. Each experiment was carried out in triplicate.

NIH3T3 cells were serum starved for 24 h to examine whether inositol deficiency affected ciliogenesis. Ciliated cells were visualized by confocal microscopy (Figure 5A). The number of total cells and ciliated cells were counted and percentage of ciliated cells was calculated. More than 65% of control group cells formed primary cilia, and only 20% of Li_2_CO_3_ treated cells formed primary cilia (Figure 5B). The length of primary cilia in Li_2_CO_3_ treated cells was on average~40% shorter than that in controls (Control group=2.1 ± 0.5 μm; Li_2_CO_3_ group=1.2 ± 0.3 μm; Figure 5C, *P* < 0.01). Scanning electron microscopy showed data consistent with the results of immunofluorescence (Figure 6). Most control cells formed primary cilia, with normal size, extending from the cell surface (Figure 6A). However, much fewer cells formed primary cilia after treatment with Li_2_CO_3_; some cells only had small protrusions from the cell surface, and some formed primary cilia with shorter lengths (Figure 6A). Statistical analysis revealed significant differences both in the number and length of primary cilia between the two groups (Figures 6B,C, *P* < 0.01). Collectively, our results suggested that suppression of *Inpp5e* expression might disrupt ciliogenesis under inositol deficiency.

### Changes in Concentrations of Inositol and Its Metabolites in Mouse and Cell Models

To confirm the inhibitory effect of Li_2_CO_3_ on inositol biosynthesis *in vivo*, the dynamic changes of plasma inositol levels of pregnant mice was measured at 0, 4, 8, 16, 24, and 48 h after Li_2_CO_3_ (350 mg/kg) injection at E 7.5, using an established GC-MS method. As shown in Table 3, plasma inositol levels decreased continuously from 0 h to 8 h after Li_2_CO_3_ intervention, and reached a minimum level at 8 h (*P* < 0.01). The plasma inositol level began to rise from 8 to 16 h, however, it still maintained at lower levels from 16 to 48 h, compared to the basic level of 0 h. In the *in vitro* studies, the level of inositol in Li_2_CO_3_ treated NIH3T3 cells was 42.51 ng/1 x 10^6^ cells, which was significantly lower as compared with the control cells (45.61 ng/1 x 10^6^cells, Figure 4C). Previous studies have confirmed that INPP5E has enzyme activity to convert PtdIns(3,4,5)P3 into PtdIns(3,4)P2 (27). We therefore also measured the amount of PtdIns(3,4)P2. As shown in Figure 4F, the level of PtdIns(3,4)P2 was much lower in Li_2_CO_3_ treated NIH3T3 cells (*P* < 0.01). These data indicated that Li_2_CO_3_ effectively inhibited the biosynthesis of inositol and disrupted the inositol metabolic pathways.

**Table 3:** Dynamic changes of plasma inositol concentration in pregnant mice after Li_2_CO_3_ (350 mg/kg) treatment.

**Time after Li_**2**_CO_**3**_treatment (h)**	**Inositol concentration (mg/L)**
0	47.5 ± 1.59
4	30.9 ± 2.13^**^
8	21.5 ± 1.19^**^
16	39.9 ± 1.67^*^
24	31.9 ± 0.89^**^
48	28.9 ± 1.39^**^

* *P < 0.05*,

** *P < 0.01 vs. 0 h*.

### Abnormal Expression of Cilia-Related Genes in the Inositol Deficiency Mouse and Cell Models

**Figure 7:**
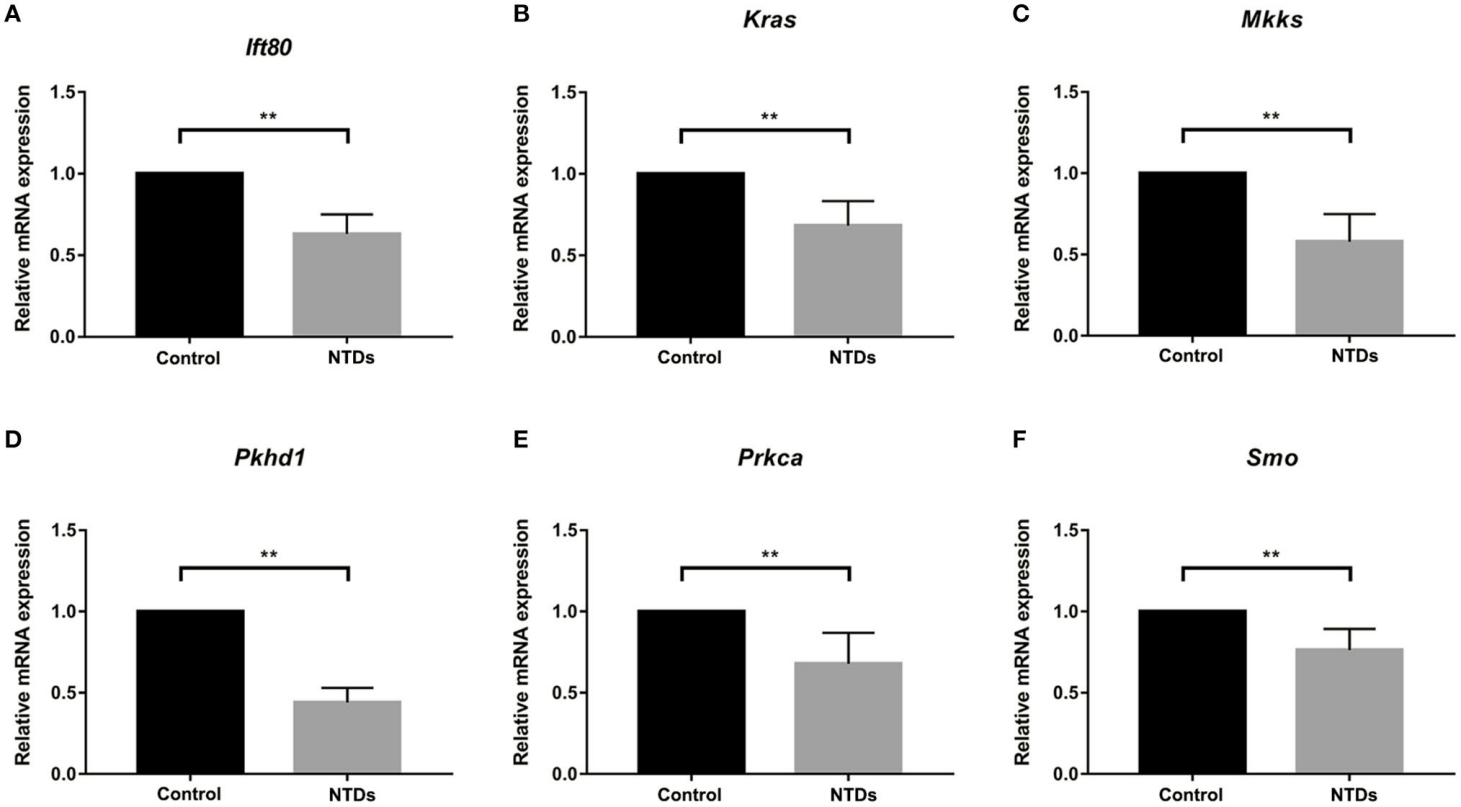
Abnormal expression of cilia-related genes in the inositol deficieny mouse model. **(A–F)** The mRNA expression of *Ift80, Kras, Mkks, Pkhd1, Prkca, Smo* in embryonic brain tissues in control (*n* = 3) and NTDs groups (*n* = 3) at E 13.5. **P < 0.01 vs. control group.

**Figure 8:**
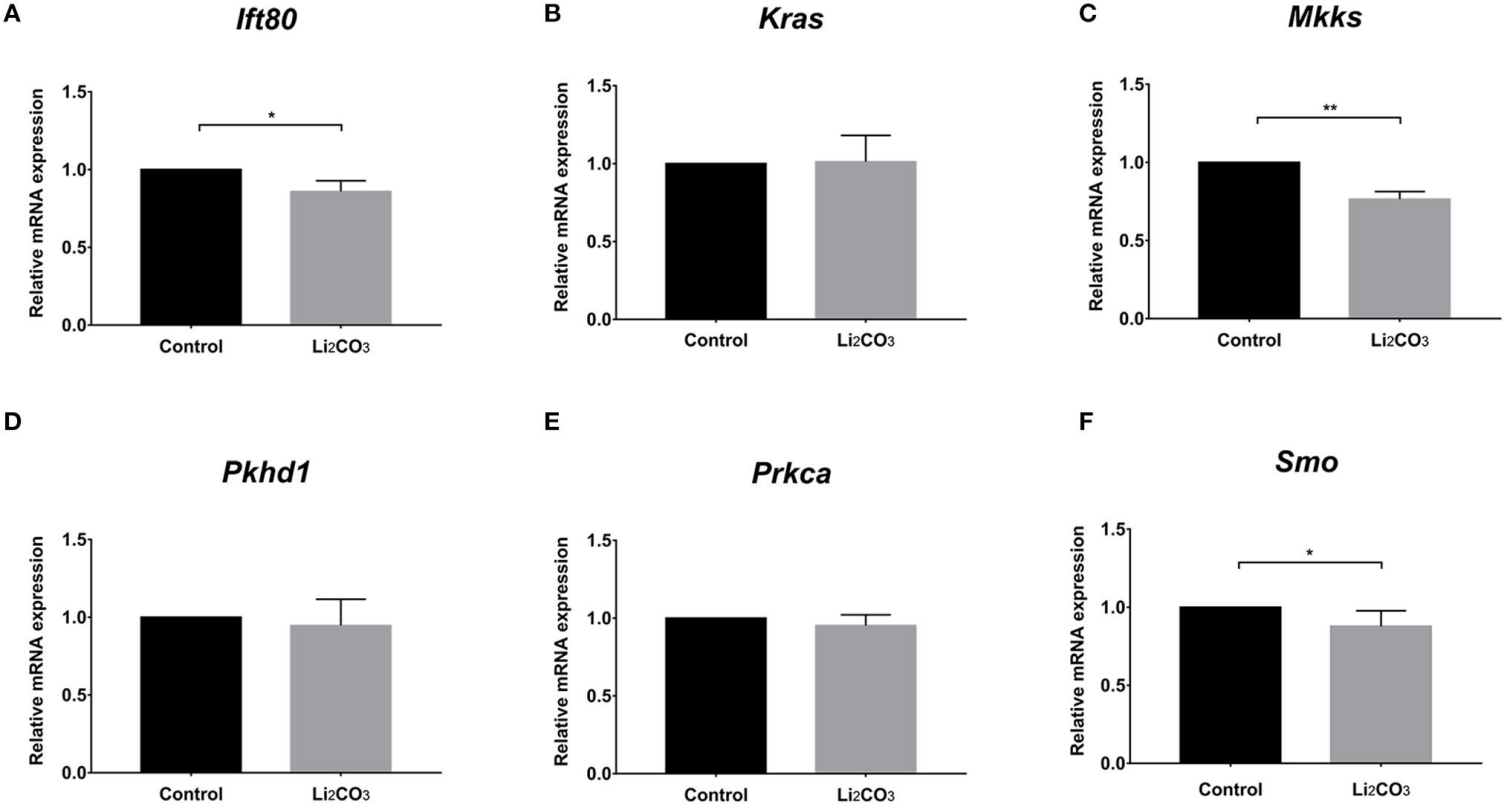
The mRNA expressions of the cilia-related genes in the inositol deficieny cell model. **(A–F)** The mRNA expressions of *Ift80, Kras, Mkks, Pkhd1, Prkca, Smo* in NIH3T3 cells treated with or without 2 mM Li_2_CO_3_ for 24 h. All the analysis in NIH3T3 cells were performed in three independent experiments (*n* = 3). Each experiment was carried out in triplicate. *P < 0.05, **P < 0.01 vs. Control group.

To further investigate whether primary cilia formation was affected during neural development in the inositol-deficient mouse model, we analyzed the expression of 89 cilia-related genes in embryonic murine brain tissues. As shown in Figure 7, there were 6 related genes significantly down-regulated in the NTDs group, compared with the control group (*P* < 0.01), which included Intraflagellar Transport 80 (*Ift80*), Kirsten rat sarcoma viral oncogene homolog (*Kras*), McKusick-Kaufman Syndrome (*Mkks*), Polycystic Kidney and Hepatic Disease 1 (*Pkhd1*), Protein Kinase C Alpha (*Prkca*) and Smoothened (*Smo*). *Ift80, Mkks, Pkhd1* and *Smo* have been demonstrated to play an important role in cilium assembly (28–31). *Prkca* and *Smo* are highly involved in the development of NTDs (32). To validate the link between the candidate cilia genes found in the mouse model and the cilia phenotype observed in cells, the mRNAs from the 6 down-regulated genes were analyzed in NIH3T3 cells. RT-qPCR revealed that the expression of *Ift80, Mkks* and *Smo* were significantly decreased in the inositol deficiency cells, compared with the control cells (Figure 8). These results indicated that these important cilia-related genes were affected in inositol-deficient conditions, which might disrupt the formation of primary cilia, and eventually lead to NTDs.

## Discussion

The *Inpp5e* gene plays a crucial role in modulating phosphoinositide homeostasis and has been implicated in the processes of mammalian neurulation and ciliogenesis. In the present study, we observed a different expression pattern of *Inpp5e* in the forebrain and hindbrain of the normal and NTDs embryos, from E 10.5 to 13.5. Furthermore, the expression of *Inpp5e* was markedly decreased in the brain tissue of a NTDs mouse model with inositol deficiency, which was consistent with the study in NIH3T3 cells cultured under conditions of inositol deficiency. Moreover, abnormal expression of cilia-related genes in embryonic murine brain tissue, and defective ciliogenesis in NIH3T3 cells were observed in the context of inositol deficiency. These results indicated that inositol deficiency affected the *Inpp5e* expression patterns and levels, which resulted in abnormal expression of the important cilia-related genes, impairing ciliogenesis, and ultimately leading to NTDs.

Many studies have suggested a non-negligible role of *Inpp5e* during the stage of neural tube closure. Cranial NTDs have occurred in mice embryos lacking *Inpp5e* (6). A null allele of *Inpp5e* caused abnormal Shh response during the developing neural tube at E 10.5 (7). Using a mouse model of NTDs with folate metabolic disturbance, we observed a gradual decline in the *Inpp5e* expression along a normal embryonic development course at E 11.5, E 13.5, E 15.5, but not in NTDs embryos; and that *Inpp5e* expression was significantly lower in NTDs embryos than controls at E 11.5 (8). In addition to folic acid, studies have shown that maternal inositol deficiency during pregnancy also contributed to the development of NTDs in the offspring (32). Hence, in this study, we analyzed the expression patterns of *Inpp5e* in the embryonic brain tissues daily for 4 consecutive days from E 10.5 to E 13.5, in forebrain, and hindbrain respectively, in a mouse model of inositol-deficiency induced NTDs. Consistent with previous studies, we demonstrate that *Inpp5e* expression in normal embryonic brain tissues exhibited a downward trend from E 11.5 to E 13.5. Furthermore, our results showed that there is a steadily decreasing trend in the expression of *Inpp5e*, in the forebrain, from E 10.5 to E 13.5, which suggests that dynamic changes in *Inpp5e* expression may play an important role in the development of the forebrain. This finding coincides with the view that primary cilia are crucial for forebrain development (33). While not due to an observable obvious trend, an increasing peak from E 11.5 to E 12.5 was seen in the forebrain of the NTDs group. There was also an increasing peak of *Inpp5e* expression in the hindbrain of control group from E 10.5 to E 13.5, while no obvious trend was observed in the hindbrain of the NTDs group. These phenomena were probably due to the precise spatial-temporal specificity of gene expression during embryonic neurodevelopment (34). Notably, in the inositol-deficient mouse model, INPP5E was found to be markedly diminished among NTDs embryos compared with the controls at E 10.5, E 11.5, E 12.5, and E 13.5. However, our previous study demonstrated that the obvious reduced expression of *Inpp5e* was observed only at E 11.5 in the folate metabolic disturbance mice model (8). These data suggest that *Inpp5e* gene might play a distinct role in the processes of neural tube closure under different nutritional status.

Many studies have shown that *Inpp5e* is required for ciliogenesis and cilia maintenance (5, 35). Mutations in the *Inpp5e* gene cause ciliopathic phenotypes such as JBTS, which is characterized by neural tube defects and polydactyly (36). Fibroblasts derived from patients with a homozygous variant in *Inpp5e* have revealed that a significant number of cells have shorter or no cilia (5). A previous study has shown that INPP5E is crucial for establishing basal restriction of ciliary structure dynamics (37). At the centrosome/ciliary base, INPP5E coordinates PtdIns(4)P homoeostasis, which is essential for ciliogenesis through modulating the centrosomal protein 164 (CEP164)-dependent recruitment of tau tubulin kinase 2 (TTBK2) (22). Consistent with previous studies, we found that the length and amount of primary cilia were significantly decreased in inositol-deficient cells, along with down-regulated *Inpp5e* expression. As a result, our study suggests that abnormal expression of *Inpp5e* might interrupt the formation of primary cilia under conditions of inositol deficiency.

**Figure 9:**
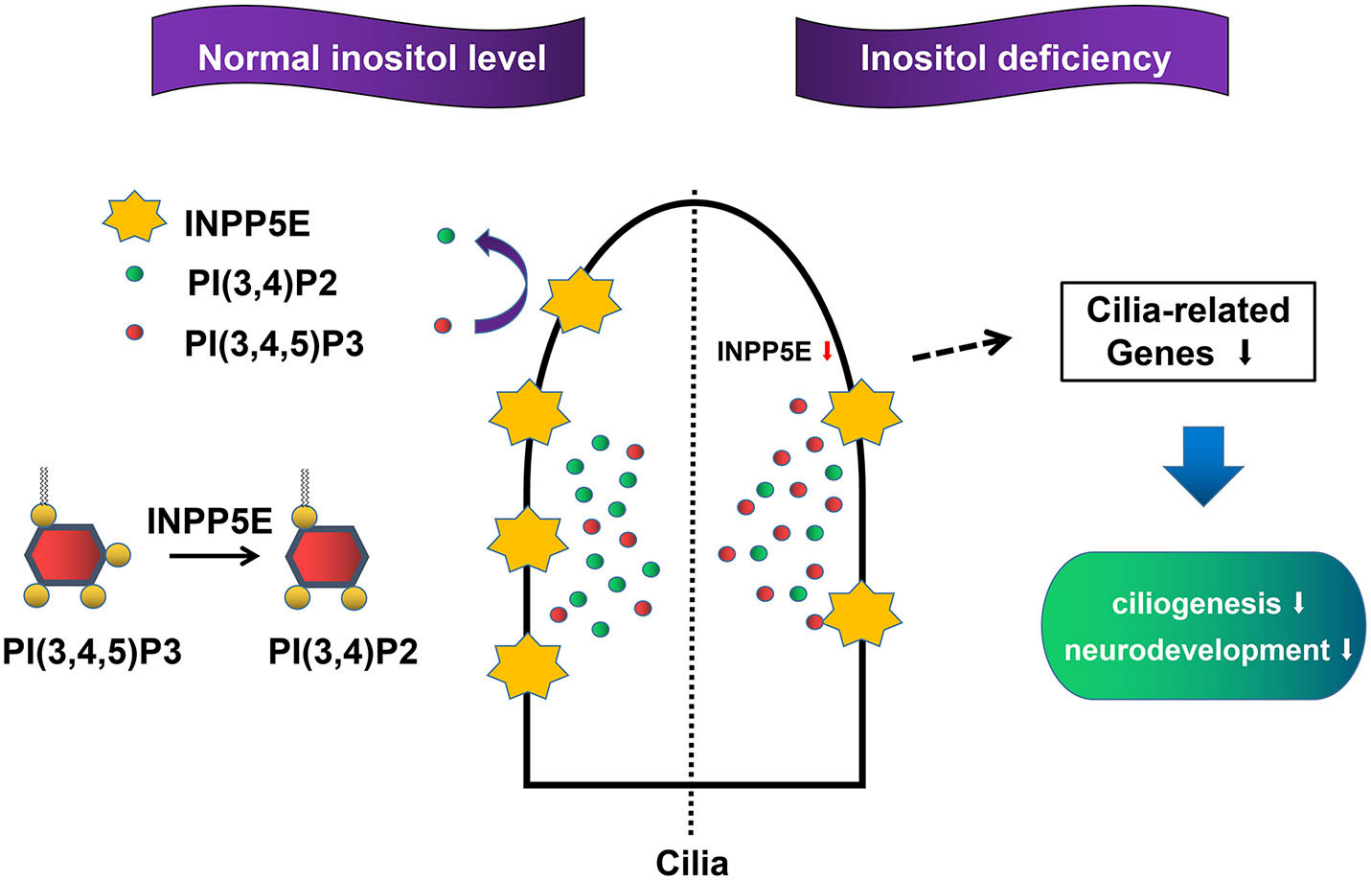
Proposed model for the role *Inpp5e* in ciliogenesis through phosphoinositide under inositol deficiency.

The primary cilia, a sensory organelle presented in most mammalian cells, play important roles in the process of embryonic development (38, 39). Growing evidence has demonstrated that primary cilia are crucial for neurogenesis, early patterning, neuronal maturation and survival, mainly through modulating cell cycle progression, Wnt signaling, and Hedgehog signaling during embryonic neural development (40–45). NTDs were observed in some mouse mutants with impaired primary cilia (17, 46). We found that six important cilia-related genes were significantly down-regulated in the inositol-deficiency mouse model, including *Ift80, Kras, Mkks, Pkhd1, Prkca*, and *Smo*. Five of these genes have been demonstrated to play an important role in cilium assembly and two genes (*Prkca, Smo*) are highly involved in NTDs (28–32). Messanger RNA expression from *Ift80, Mkks*, and *Smo* genes was significantly down-regulated in inositol deficiency NIH3T3 cells, thus strengthening the link between the cilia phenotype observed in cells with the candidate cilia genes found in the mouse model. *Ift80* gene encodes IFT80 protein, which helps carry materials from the base to the tip of cilia and is essential for the development and maintenance of primary cilia (47). *Mkks* plays a role in the assembly of BBSome, a complex involved in ciliogenesis regulating transport vesicles to the cilia (48). The Hedgehog signaling pathway is essential for embryonic development (40, 49). Smo, a G protein-coupled receptor, transduces signals to the downstream targets after activation by a hedgehog protein. It has been reported that after activation of the Hedgehog signaling pathway, accumulation of Smo at the primary cilia were found in the wild-type mice, whereas the ciliary enrichment of smo was highly reduced in *Inpp5e* null mice (50). Consequently, the results in embryonic murine brain tissue and NIH3T3 cells demonstrated that the reduced expression of *Inpp5e* might disrupt the formation of the primary cilia, and eventually lead to neurodevelopmental abnormality in inositol deficient mice (Figure 9). The exact mechanism of *Inpp5e* expression in regulating ciliogenesis under the inositol deficient condition needs to be further studied.

## Conclusion

In summary, our results suggest that down-regulation of *Inpp5e* might be associated with abnormal ciliogenesis during embryonic neurodevelopment, under conditions of inositol deficiency. This study provided a novel insight to explore the mechanism of neuromalnutrition.

## Data Availability Statement

The raw data supporting the conclusions of this article will be made available by the authors, without undue reservation.

## Ethics Statement

The animal study was reviewed and approved by Ethics Committee of Capital Institute of Pediatrics.

## Author's Note

RZ is the senior author for the molecular biology research group.

## Author Contributions

All the authors contributed considerably to this study. HY, SL, and TG performed the experiments and drew the figures. XW and ZG analyzed the data. JQ provided reference materials and arranged the manuscript in accordance with the journal specifications. BN and RZ contributed reagents and analysis tools. YL and ZZ searched and provided material for writing the manuscript. HY wrote the manuscript. JW and JG designed the experiments. JW, JG, and JL revised and approved the manuscript. All authors read and approved the manuscript for submission.

## Conflict of Interest

JL was employed by company InnoStar Bio-tech Nantong Co., Ltd., Nantong, China. The remaining authors declare that the research was conducted in the absence of any commercial or financial relationships that could be construed as a potential conflict of interest.
